# Paul S. Martin (1928–2010): Luminary, Natural Historian, and Innovator

**DOI:** 10.1371/journal.pbio.1001016

**Published:** 2011-02-08

**Authors:** C. Josh Donlan, Harry W. Greene

**Affiliations:** 1 Advanced Conservation Strategies, Midway, Utah, United States of America; 2 Department of Ecology and Evolutionary Biology, Cornell University, Ithaca, New York, United States of America

**Figure:**
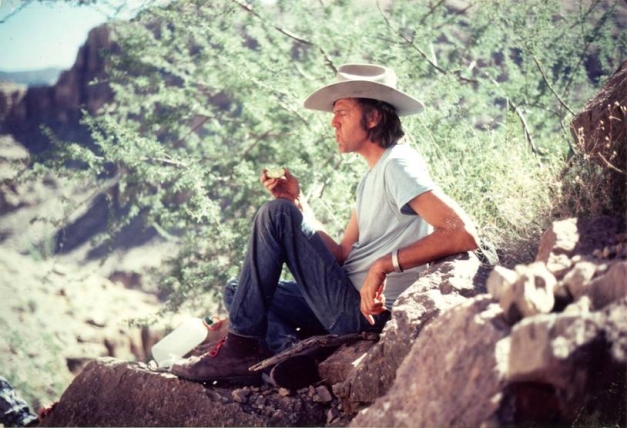
Paul Martin at Rampart Cave, home of the Shasta Ground Sloth, Grand Canyon, ca. 1975. Photo: Thomas R. Van Devender.

*To behold the Grand Canyon without thoughts of its ancient condors, sloths, and goats is to be half blind*—Paul S. Martin 1992

Paul S. Martin, a monumental figure in paleontology and ecology, died on September 13, 2010, at the age of 82. He wanted others to know, conveyed in a message from his wife, that when the time came, he would “die” and then be “dead.” He would not pass on, go gently, meet his maker, or go over to the other side. Paul combined an intense love for life, adventure, and natural history with innovative thinking, to an extent that is increasingly rare in today’s age of specialization. His impact on science was deep and transformative, cutting across many disciplines including ecology, paleontology, anthropology, and biodiversity conservation.

Paul is best known for his work on the loss of America’s charismatic megafauna, from mastodons and mammoths to saber-toothed cats and sloths. He first published on the subject in 1958, and later authored seminal works on the subject [1]–[5]. Early in his career, Paul framed what has become one of the most enduring scientific whodunits of all time: what drove Late Pleistocene extinction—and what role did humans play? In 1966, writing about his “overkill hypothesis,” in which he indicted early humans for Pleistocene extinctions, Paul commented that it “is likely to continue to provoke serious and perhaps unanswerable objections” [5]. That turned out to be an understatement: the role of humans in Pleistocene extinction has been debated, often heatedly and almost continuously, for the last 50 years. While Paul relentlessly promoted the idea humans played the significant role in the demise of the Pleistocene megafauna, he embraced even his most critical dissenters—indeed, he invited criticism. His colleague Karl Flessa commented, “Paul really wanted to see things the way his opponents saw them, in order to understand even more about his favorite topic, Pleistocene extinctions." Paul’s 50-year journey with Pleistocene extinction is documented in his memoir *Twilight of the Mammoths* [6].

But Paul’s impact on science goes far beyond Pleistocene extinction. Paul Martin was a true Renaissance man—leveraging natural history to innovate new perspectives time and time again. In his monograph *The Last 10,000 Years: A Fossil Pollen Record of the American Southwest*, Paul took palynology (the study of pollen grains and other spores) to a far from obvious place: the dry lakes of Arizona. Edward Deevey, Paul’s post-doctoral supervisor, declared, “His pioneering work is exciting, not only for what it tells, but even more for what it promises: history, free of peat-bound preconceptions, in a land of little mud” [7].

Left wanting to unravel more tales of the ecological past, Paul then turned to packrat middens, helping to develop a new subdiscipline of paleontology that is among the most important tools of historical ecology [8]. His classic publication, *Neotropical Anachronisms* with Dan Janzen, changed the way ecologists view species interactions by elucidating the role of extinct vertebrates on contemporary ecology and life history traits [9]. Paul had a life-long love affair with Mexico and the country’s diverse flora and fauna. He spearheaded the Rio Mayo Project, updating Howard Scott Gentry’s *Rio Mayo Plants: A Study of the Flora and Vegetation of the Valley of the Rio Mayo, Sonora* [10], a project that involved 20+ years of field trips and the collection of over 15,000 specimens. From remote mangroves to ridge top conifer forests in the Sierra Madre Occidental, Paul traveled across one of Mexico’s most precious and inaccessible biomes—by bush plane, vehicle, burro, kayak, and foot. This was a remarkable feat for any biologist, even more extraordinary for a man that suffered from polio early in life and later relied on crutches.

Born in Allentown, Pennsylvania, Paul earned his bachelor’s degree in zoology from Cornell University. From his teens through his eighth decade, Paul was all about fieldwork. By the time he graduated from Cornell in 1951, he had published papers on the natural history of bicolored hawks and black robins in western Mexico. Paul told us of grand times shooting birds for museum specimens with a shotgun out of the top floor of Cornell’s Fernow Hall, and of long road trips to the cloud forests of Tamaulipas, Mexico that involved an old jeep, binoculars, bad roads, and lots of flat tires. In those days, the 2000+-mile trip to Tamaulipas was likely worth a degree in itself. He went on to earn a Master’s and doctorate from the University of Michigan, studying the biogeography of amphibians and reptiles in those forests, an exercise in historical ecology that heavily influenced his life. Paul spent four years at Yale University and the University of Montreal as a post-doctoral researcher before moving to the Geochronology Laboratory at the University of Arizona in 1957. He would enjoy the rest of his career in Tucson, much of it as a professor of geosciences at the University’s Desert Laboratory, perched on Tumamoc Hill.

Aside from his contributions to paleontology and ecology, Paul also influenced how we think about biodiversity conservation. For decades, almost single-handedly, he pondered the impact of megafaunal loss during the end of the Pleistocene on contemporary ecosystems: “Perhaps the long-lauded home where buffalo roam,” he wrote in 1969, “is also the land where camel and eland should play” [11]. His insights on the role of ecological history in biodiversity conservation culminated with the recent proposal of Pleistocene Rewilding, a call for science-based restoration of missing ecological functions and evolutionary potential of lost megafauna using extant conspecifics and related taxa [12],[13]. Paul was keenly aware that nativeness, place, and history are central to the science, strategies, and aesthetics of biodiversity conservation. His work was central in elucidating the all-to-common post-Columbian bias that blinds a paleoecological view of biodiversity and ecosystems.

Another influential ecologist, Larry Slobodkin, died a year and a day before Paul Martin. Late in life Slobodkin was asked, “What do ecologists do?” He replied that it consists of choosing one of three paths and doing it well: the first was to become an expert on some group of organisms that excites you; the second was to master the most cutting-edge techniques; and the third, the most perilous path, was to “strenuously avoid doing what everyone else is doing and search for new ideas and new tests for old ideas” [14]. Paul Martin remarkably did all three brilliantly. The mystery of the Pleistocene extinction fascinated the naturalists of the 18th and 19th century, including Darwin, Wallace, Lyell, Owen, and Cuvier. Paul contributed more to solving this grand enigma than anyone living or dead. He was a pioneer in interdisciplinary research long before it was popular on academic campuses, and he sought new perspectives in natural history, often seizing on novel technologies to solve problems. But, Paul was more than that: he loved life and loved people. While our interactions with Paul were largely restricted to his later years, his influence on us—like many—was transformative. He even loved chiggers, harbingers of summer rain. One morning over coffee in the Arizona hill country, Paul declared with a big grin, “May the gods smile, the chiggers are rampant in the green hills of Sonoita these days.”
